# Androgen receptor expression predicts different clinical outcomes for breast cancer patients stratified by hormone receptor status

**DOI:** 10.18632/oncotarget.9778

**Published:** 2016-06-07

**Authors:** He-Sheng Jiang, Xia-Ying Kuang, Wei-Li Sun, Yan Xu, Yi-Zi Zheng, Yi-Rong Liu, Guan-Tian Lang, Feng Qiao, Xin Hu, Zhi-Ming Shao

**Affiliations:** ^1^ Key Laboratory of Breast Cancer in Shanghai, Department of Breast Surgery, Fudan University Shanghai Cancer Center, Shanghai, China; ^2^ Department of Oncology, Shanghai Medical College, Fudan University, Shanghai, China; ^3^ Institutes of Biomedical Science, Fudan University, Shanghai, China; ^4^ Department of Pathology, Fudan University Shanghai Cancer Center, Shanghai, China; ^5^ Department of Breast Surgery, The First Affiliated Hospital, Sun Yat-Sen University, Guangzhou, China

**Keywords:** breast cancer, TNBC, androgen receptor, BRCA1, hormone receptor

## Abstract

In this study we sought to correlate androgen receptor (AR) expression with tumor progression and disease-free survival (DFS) in breast cancer patients. We investigated AR expression in 450 breast cancer patients. We found that breast cancers expressing the estrogen receptor (ER) are more likely to co-express AR compared to ER-negative cancers (56.0% versus 28.1%, *P* < 0.001). In addition, we found that AR expression is correlated with increased DFS in patients with luminal breast cancer (*P* < 0.001), and decreased DFS in TNBC (triple negative breast cancer, *P* = 0.014). In addition, patients with HR+ tumors (Hormone receptor positive tumors) expressing low levels of AR have the lowest DFS among all receptor combinations. We also propose a novel prognostic model using AR receptor status, BRCA1, and present data showing that our model is more predictive of disease free survival compared to the traditional TMN staging system.

## INTRODUCTION

Breast cancer is a heterogeneous disease with several distinct subtypes that are based on differential patterns of gene expression. Such a heterogeneity leads to markedly different treatment approaches and outcomes, which in turn necessitates a deeper understanding of prognostic and predictive markers. In breast cancer, the oncogenic roles of nuclear steroid hormone receptor (HR) signaling mediated by the estrogen receptor (ER) and progesterone receptor (PR), respectively, have been extensively characterized. The findings provide the basis for receptor antagonist therapy today. In contrast, the roles of androgen receptor (AR), which is highly expressed in all breast cancers (60-70%) regardless of ER status [1, 2], remains less clearly defined. AR interacts with the ER signaling pathway [1, 3], thereby making it an attractive therapeutic target and, likely, a prognostic marker. Previous studies hypothesized AR as a good prognostic marker in ER^+^ tumors, but portends a poor prognosis in ER^−^ tumors [4]. However, clinical data supporting the hypothesis is unconvincing, AR has been reported as a favorable prognostic factor in ER^+^ breast cancer [4–6], but its prognostic value remains controversial in ER^−^ tumors [7–9]. Triple negative breast cancer (TNBC) lack ER and PR expression as well as human epidermal growth factor receptor 2(HER2). Patients with TNBC have significantly worse prognosis compared to other breast cancer subtypes due to the lack of well-defined targeted molecular therapy. Multiple TNBC subtypes have been described, among which is basal-like subtype(BL1 and BL2) that harbors mutation of the breast cancer-associated gene 1(BRCA1), which was discovered as the first breast cancer susceptibility gene in familial breast cancer [10], and is defective in DNA repair [11], and the luminal androgen receptor subtype (LAR) which expresses AR. Patients with LAR have decreased relapse-free survival [11]. In this study, we profile the expression pattern of AR in 450 patients, ranging from stage I to III based on Tumor, Node and Metastasis (TNM) staging system, and we correlated AR expression with clinical outcome to evaluate its prognostic implication alone or in combination with HR status. Furthermore, we constructed a prognostic model combining AR and BRCA1 with the traditional model, to provide a more sensitive and specific method for predicting survival in TNBC.

## RESULTS

### Patient characteristics

A total of 450 cases of primary invasive breast cancer were included in this cohort. Twenty-eight cases were censored for lack of follow-up, and AR expression could not be measured by IHC in 16 cases because of tissue core loss. Thus, 407 cases were included in the subsequent analyses. The clinical-pathological features of this cohort are summarized in Table 1. All patients were female with a mean age of 51.31 years at diagnosis. Ninety-two patients experienced disease recurrence during the follow-up period.

**Table 1:** Characteristics of breast cancer patients

Clinical-pathological	cases	AR		
characteristics		AR^low^	AR^high^	*p* value^α^
		(% of total)	(% of total)	
**Age (years)**				
≤50	226(51.7)	141(32.3)	85(19.5)	0.382
>50	211(48.3)	123(28.1)	88(20.1)	
**Menopausal status**				
Pre	224(51.3)	129(29.5)	95(21.7)	0.216
Post	213(48.7)	135(30.9)	78(17.8)	
**TNM stage**^b^				
I	133(31.4)	74(17.5)	59(13.9)	0.222
II	241(56.8)	144(34.0)	97(22.9)	
III	50(11.8)	36(8.5)	14(3.3)	
**Pathologicalstage**^b^				
I	7(1.7)	3(0.7)	4(1.0)	0.622
II	293(70.8)	176(42.5)	117(28.3)	
III	114(27.5)	70(16.9)	44(10.6)	
**Tumor size (cm)**				
T1(≤2)	202(47.1)	115(26.8)	87(20.3)	0.163
T2 (>2_5)	206(48)	129(30.1)	77(17.9)	
T3 (>5)	21(4.9)	16(3.7)	5(1.2)	
**Node status**				
Negative	244(56.1)	141(32.4)	103(23.7)	0.745
Positive	191(43.9)	122(28.0)	69(15.9)	
**ER status**				
Negative	249(57.5)	179(41.3)	70(16.2)	**<0.01**
Positive	184(42.5)	81(18.7)	103(23.8)	
**PR status**				
Negative	293(67.8)	201(46.5)	92(21.3)	**<0.01**
Positive	139(32.2)	58(13.4)	81(18.8)	
**HER2 status**				
Negative	255(58.9)	150(34.6)	105(24.2)	0.459
Positive	178(41.1)	111(25.6)	67(15.5)	
**Anthracyclines based chemo**				
negative	90(20.6)	50(11.5)	40(9.2)	0.359
Positive	346(79.4)	213(48.8)	133(30.5)	
**Taxane based chemo**				
negative	388(89.0)	238(54.6)	150(34.4)	0.216
Positive	48(11)	25(5.7)	23(5.3)	
**Local recurrence**				
Negative	354(90.8)	205(52.6)	149(38.2)	0.337
Positive	36(9.2)	23(5.9)	13(3.3)	
**Distant metastasis**				
Negative	316(81.0)	176(45.1)	140(35.9)	**0.022**
Positive	74(19)	52(13.3)	22(5.6)	

Abbreviations: ER: estrogen receptor; PR: progesterone receptor; HER2: human epidermal growth factor receptor 2; AR: androgen receptor

Bold values are significant (*P* < 0.05).

α Compared using Student's t test or Pearson's χ2 test.

b Classified according to the National Comprehensive Cancer Network guidelines.

Significant differences in AR expression patterns were observed among the different breast cancer subtypes

The TMAs were stained for AR (Figure S1) and BRCA1 (Figure S2) using previous published methods [16]. AR expression was measured in 434 cases: 172 cases (39.6%) were AR high-expressed, 76 cases (17.5%) were AR low-expressed, and 186 cases (42.9%) were AR negative. Significant differences in AR expression patterns among different breast cancer subtypes are observed. The rate of high AR expression was 52.3% in the luminal subtype, 34.4% in the HER2-positive subtype, and 25.7% in TNBC (Figure S3, Table S1). AR expression correlated positively with ER (*P* < 0 .001) and PR (*P* < 0.001) status but negatively with metastasis (*P* = 0.022). However, no significant correlation was observed with the other tumor characteristics (Table 1).

### Androgen receptor expression was associated with different prognostic outcomes for breast cancer patients stratified by joint hormone receptor status

**Figure 1:**
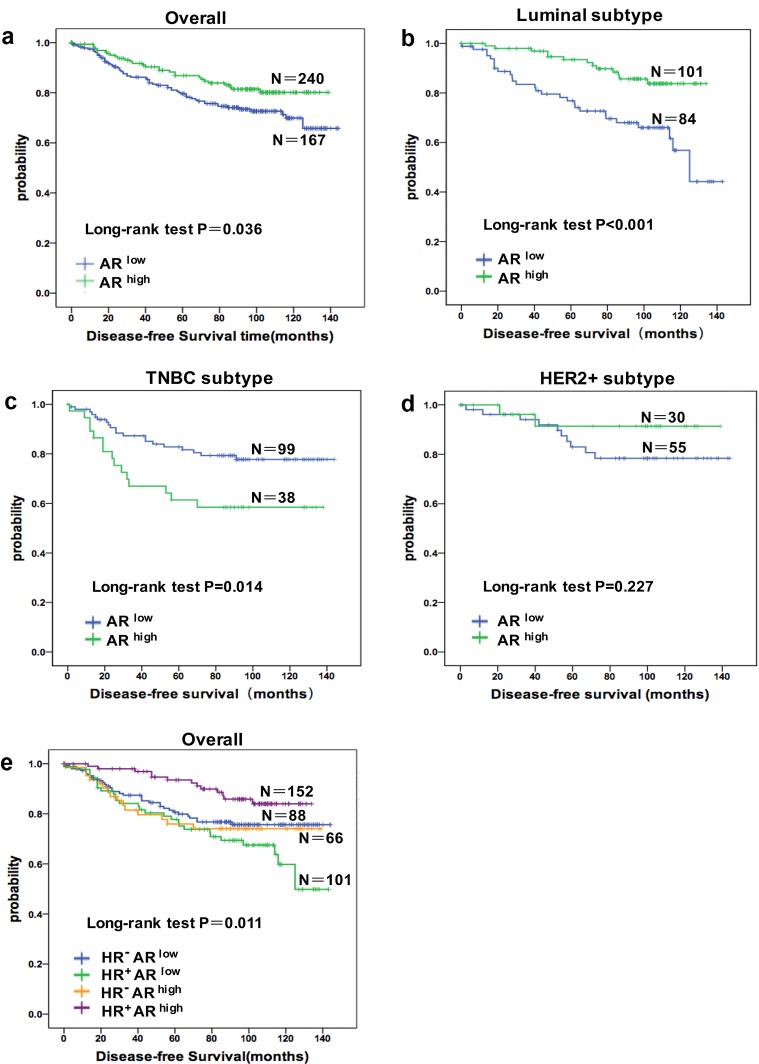
The prognostic role of AR alone in different population and stratified by joint hormone receptor (HR) status Cumulative Disease-free Survival(DFS) curves of **a.** all patients (*n* = 407), **b.** Luminal subtype patients (*n* = 185), **c.** TNBC patients (*n* = 137), **d.** HER2 positive patients (*n* = 85), **e.** combinations of AR and HR status.

Patients were followed for up to 144 months (median follow-up time = 91.0 months). Four hundred and twenty-two patients completed the follow-up, and 92 events were observed. In general, patients with AR^high^ tumors had a significantly favorable prognosis compared with patients with AR^low^ tumors (Figure 1a). This prognostic significance was consistent in the luminal subtype (Figure 1b) but inconsistent in TNBC, in which patients with AR^high^ tumors had a worse prognosis (Figure 1c). Stratification by HR status revealed that AR was a positive prognostic marker in patients with HR^+^ (ER or PR positive) tumors but conferred a worse prognosis in patients with HR^−^ tumors (both ER and PR negative). Combining HR and AR status revealed a worse prognosis for patients with HR^+^AR^low^ tumors but a superior prognosis for those with HR^+^AR^high^ tumors compared with all other combinations (Figure 1e). Tumors with discordant ER and AR status (HR^+^AR^low^ or HR^−^AR^high^) were associated with a worse prognosis compared with tumors with concordant ER and AR status (HR^−^AR^low^ or HR^+^AR^high^).

### Development of a prognostic signature using combined Androgen receptor and BRCA1 status for TNBC patients

**Figure 2:**
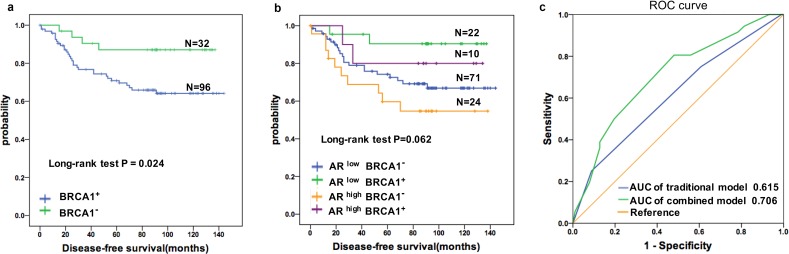
Prognostic value of the AR in TNBC patients was improved by combining BRCA1 status **a.** Cumulative Disease-free Survival(DFS) curves of TNBC patients by BRCA1 status. **b.** Kaplan-Meier estimates of DFS according to AR and BRCA1 statuses in TNBC patients (log rank *P* = 0.062) **c.** ROC curves assessing the distinct performances of the combined and the traditional models for predicting the DFS in the TNBC cohort. Variables for the traditional model include TNM stage only. AR and BRCA1 were added in the combined model. *p* < 0.001 for AUC comparison.

When the correlations between DFS in patients with TNBC and each clinical-pathological variable were examined by univariate analysis, several factors demonstrated a significant association (Table 2). Positive lymph node status (HR = 2.262; 95% CI 1.175-4.356; *P* = 0.015), tumor size > 5 cm (HR = 1.712; 95% CI 1.031-2.842; *P* = 0.038), and stage III cancer (HR = 2.111; 95% CI 1.250-3.566; *P* = 0.005) were associated with a higher risk of disease relapse. Moreover, elevated AR expression indicated a higher risk of disease relapse (HR = 2.258, 95% CI 1.155-4.414; *P* = 0.017), whereas higher BRCA1 expression was associated with a lower risk of disease events (HR = 0.321; 95% CI 0.113-0.908; *P* = 0.032); these findings were consistent with a Kaplan-Meier analysis demonstrating that nuclear BRCA1 expression was associated with a favorable prognosis in patients with TNBC (Figure 2a). Moreover, the DFS of patients with AR^high^BRCA1^−^ tumors was significantly worse than that for patients in other subgroups (Figure 2b). A stepwise multivariate Cox model was used to examine traditional clinical parameters that are prognostic factors for DFS in TNBC (Table 2). Only TNM stage was identified as a dominant prognostic factor for DFS (*P* < 0.01). Thus, the traditional model (M_traditional_) was as follows:

$${\text{M}}_{\text{traditional}}=0.446\times \text{TNM stage}$$

**Table 2:** Univariate and multivariate analysis of factors related to DFS in TNBC cancer patients

	DFS			
variable	Univariate analysis		Multivariate analysis	
	HR (95 % CI)	*p* value	HR (95 % CI)	*p* value
**age**	1.471(0.752-2.876)	0.259		
**TNM stage**	**2.111(1.250-3.566)**	**0.005**	**2.263(1.085-4.723)**	0.03
**tumor size**	**1.712(1.031-2.842)**	**0.038**	1.387(0.784-2.452)	0.261
**node status**	**2.262(1.175-4.356)**	**0.015**	1.154(0.489-2.727)	0.744
**Pathological stage**	1.338(0.688-2.601)	0.391		
**Menopausal status**	1.740(0.881-3.435)	0.111		
**BRCA1**	**0.321(0.113-0.908)**	**0.032**	**0.318(0.110-0.918)**	**0.034**
**Anthracyclines based chemo**	0.677(0.339-1.354)	0.270		
**Taxane based chemo**	1.555(0.731-3.308)	0.252		
**AR**	**2.258(1.155-4.414)**	**0.017**	**2.423(1.211-4.848)**	**0.012**

Bold values are statistically significant (*P* < 0.05)

CI confidence interval

AR androgen receptor

BRCA1 breast cancer-associated gene1

With the inclusion of AR and BRCA1, TNM stage, AR (*P* = 0.026) and BRCA1 (*P* = 0.016) were identified as significant prognostic factors in a multivariate Cox model. The combined model (M_combined_) was as follows:

$${\text{M}}_{\text{combined}}=(0.817\times \text{TNM stage})+(0.885\times \text{AR})-(1.144\times \text{BRCA1})$$

The relationship between sensitivity and the false-positive rate (1-specificity) is illustrated by a ROC curve. The AUC was 0.615 (95% CI 0.560-0.709) for the traditional model and 0.706 (95% CI 0.680-0.814) for the AR-BRCA1 combined model (Figure 2c), suggesting that combining AR and BRCA1 may provide additional prognostic value for patients with TNBC (*P* < 0.001 for the AUC comparison).

## DISCUSSION

Androgen-based therapy in breast cancer was first described in the 1940s [18], its mechanisms remain unclear. We sought to dissect the androgen signaling pathways by exploring the associations between AR and cancer; Previous studies reported that AR could have either beneficial or deleterious effects depending on the breast cancer subtype [17, 18]. We find that AR has prognostic significance alone or in combination with HR status, and our data suggest that AR is associated with improved prognosis in luminal breast cancer and worse prognosis in TNBC.

Consistent with findings in previous studies and preclinical studies [3, 7, 19], we identified AR status as a prognostic marker for DFS in patients with breast cancer. Karin Elebro et al. demonstrated that patients with breast cancer with discordant HR status (ER^+^AR^−^ or ER^−^AR^+^) had a worse prognosis compared to concordant HR status (ER^+^AR^+^ or ER^−^AR^−^), with ER^−^AR^+^ tumors being associated with the worst prognosis [7]. However, our data suggest that HR^+^AR^low^ status was associated with the worst prognosis among all combinations, potentially because of the inclusion of PR status in the definition of HR status and that we defined 45% positive nuclei cutoff as AR positive. These findings highlighted that patients with HR^+^AR^low^ tumors may have poor survival despite the luminal subtype, which is generally recognized as a favorable pathology. Therefore, more powerful adjuvant treatments should be directed to patients with HR^+^AR^low^ luminal breast cancer to prevent disease relapse.

Preclinical studies have shown that AR inhibits ER activity by blocking it's downstream transcription targets, thus inhibiting ER-stimulated tumor growth in ER-positive cell lines [3, 20]. The proposed mechanism is that ligand-activated AR translocates to the nucleus and repress ER-dependent transcription by competing for binding at ER-response elements [3]. However, in ER-negative cell lines, the opposite phenomenon is observed, cell growth, which depends on AR, was inhibited when AR was knocked down. Because AR and ER share similar ChIP-seq binding profiles [19–21], it was concluded that in the presence of ER, AR interacts with estrogen response elements, thereby blocking the expression of downstream estrogen target genes and inhibiting ER-stimulated tumor growth. In the absence of ER, AR instead binds androgen response elements and functions as an oncogene, promoting tumor growth via a separate pathway [20]. This model accounts for the opposite effects of AR status in luminal breast cancer and TNBC.

Treating TNBC has always been challenging because of the heterogeneity and the absence of well-defined molecular targets. The new discovery of promising biomarkers for TNBC will help to resolve this crisis. BRCA1 and AR are representative markers related to the basal-like and LAR subtypes of TNBC, respectively [11]. The role of AR in TNBC has been debated based on results from recent studies and underpowered to make definitive conclusions [6, 18, 20, 22]. Recently, molecular characterization efforts have pointed to AR as a potential therapeutic target for TNBC, and our data suggest that AR is inversely proportional to DFS in patients with TNBC, so there is an opportunity for AR-targeted therapies to be as effective as or better than current standard of care treatments for TNBC. Indeed, a phase II trial of bicalutamide, an androgen antagonist, in patients with metastatic AR^+^ER^−^ breast cancer, the 6-month clinical benefit rate was 19% for bicalutamide, which established the potential of targeting AR in ER- disease [23]. Another separate study using the AR signaling inhibitor enzalutamide showed improved overall survival in patients with AR+ tumors compared to patients with AR- tumors, further suggesting AR as a potential therapeutic target in TNBC (clinicaltrials.gov: NCT01889238). Moreover, there are many upcoming clinical trials exploring the utility of AR-targeted therapies (clinicaltrials.gov: NCT00468715, NCT00516542, NCT00755885, NCT00972023).

BRCA1 is famous as cancer susceptibility gene in familial breast cancer, and during preclinical research, depletion of BRCA1 impaired differentiation but promoted proliferation of mammary epithelial cells [24]. which make it reasonable that BRCA1 was positively associated with increased DFS in patients with TNBC in our study. BRCA1 plays an important role in DNA double-strand break repair, thereby contributing to the maintenance of DNA stability [25]. Poly ADP-ribose polymerase (PARP) enzymes are critical for the appropriate processing and repair of DNA breaks [26]. Preclinical studies have demonstrated that tumor cell lines lacking functional BRCA1 or BRCA2 are sensitive to PARP inhibitors [27]. Clinical trials of both PARP inhibitors and DNA-damaging agents (e.g., cisplatin) administration in *BRCA1/2*-mutant TNBC tumors have shown promising clinical benefit [28]. Therefore, PARP inhibitors could be considered a new therapeutic strategy for improving the clinical outcome of patients with TNBC that lacks BRCA1 expression.

To the best of our knowledge, our study is the first to investigate the prognostic significance of the combined BRCA1 and AR status in TNBC patients. Our model suggest that the combination of BRCA1 and AR status in TNBC prognosis is more sensitive and accurate compared to the traditional prognostic markers, potentially offering additional information for oncologists to predict patients' prognosis.

In conclusion, AR is associated with different prognosis depending on HR status, and patients with TNBC may benefit from new treatment options, such as anti-androgens or PARP inhibitors. Lastly, combining AR and BRCA1 status with traditional prognostic factors improves prognostic predictions.

## MATERIALS AND METHODS

### Patients and specimens

A total of 450 pathologically defined breast cancer samples were collected at the Department of Breast Surgery at FDUSCC (Shanghai, P.R. China) between August 2001 and January 2008. patients' enrollment process (Figure S4) and the inclusion criteria was presented in Supplementary Data. Clinical-pathological characteristics of the patients are summarized in Table 1. In this study, the patients were regularly followed, with the last update on October 31, 2014. The median follow-up time was 91.0 months (IQR 47.1-109.0).

### Breast cancer tissue microarray construction

For tissue microarrays (TMAs), samples from 207 luminal-like subtype cases, 93 HER2-enriched subtype cases and 150 TNBC cases were randomly collected from 4,179 cases that met the eligibility criteria before the initiation of cancer treatment (Figure S4). The TMAs were generated by the Department of Pathology at the FDUSCC. The TMAs construction methods was described in Supplementary Data [12, 13].

### Immunohistochemistry

Immunohistochemical staining for AR and BRCA1 was performed following a two-step protocol (GT Vision^™^ III), Which was presented in Supplementary Data.

### Staining evaluation

A senior breast pathologist (AE) who was blinded to the clinical data reviewed each TMA twice to assess the TMA section evaluation, status and invasiveness. Tumors were considered AR positive if more than 10% of the nuclei were stained, independent of the intensity [7]. A cut-off of > 45% of stained nuclei was used to define tumor with high AR expression, and tumors with between 10% and 45% stained nuclei were defined as having low AR expression. The cutoff (45%) for classification was calculated using an X-tile plot (version 3.6.1) [14]. The cases were classified into two subgroups for the statistical analyses: AR^high^ (tumors with high AR expression) and AR^low^ (AR negative tumors and those with low AR expression). For BRCA1, the TMAs were semi-quantitatively scored according to a staining index (SI; range 0-9) which was defined in Supplementary Data. SI > 5 was defined as BRCA1-positive staining, whereas SI < 5 was defined as negative staining [15]. The average score for duplicate cores was used for all subsequent analyses.

### Statistical analyses

All analyses were performed using SPSS (version 20.0; SPSS Inc.). Disease-free survival (DFS) was defined as the time from primary surgery to the date of relapse, breast cancer-specific death or October 31, 2014. A chi-squared analysis or Fisher exact test was used to test for the association between AR expression and clinical-pathological characteristics. Survival data were evaluated by Kaplan-Meier analysis. We constructed models to predict DFS in patients with TNBC using univariate and multivariate Cox analyses. Risk scores and time-dependent receiver operating characteristic (ROC) curves were calculated as previously described [15]. The area under the curve (AUC) and the 95% CI were calculated to estimate the utility of the prediction model. All P values are two-sided, and statistical significance was established at *P* < 0.05. All analyses were based on the available data, and missing data were randomly distributed.

## SUPPLEMENTARY MATERIALS FIGURES AND TABLES







## Abbreviations

AR: androgen receptor
CI: confidence interval
HER2: human epidermal growth factor receptor 2
HR: hazard ratio
HR: hormone receptor
BRCA1: breast cancer-associated gene 1
TNBC: triple negative breast cancer
PR: progesterone receptor
