# Supplementary material for: Androgen receptor expression predicts different clinical outcomes for breast cancer patients stratified by hormone receptor status
Source: Oncotarget. 2016 Jun 7;7(27):41285–93. doi: 10.18632/oncotarget.9778 (PMC5173059; doi:10.18632/oncotarget.9778)
Supplement: Supplementary file 1 [file oncotarget-07-41285-s001.pdf]

Androgen receptor expression predicts different clinical outcomes for breast cancer patients stratified by hormone receptor status

Supplementary Material

Methods

Patients and specimens

Written informed consent form was obtained from each patient participating in this study, and the institutional review board (IRB) of Fudan University Shanghai Cancer Center (FDUSCC) approved this study. Inclusion criteria of this study: (i) female patients diagnosed with stage I to III primary breast cancer; (ii) patients with unilateral invasive ductal carcinoma (IDC); (iii) patients with no proof of metastasis at diagnosis; and (iv) patients who underwent mastectomy and axillary lymph node dissection or breast conservation surgery followed by adjuvant treatment; Patients with ductal carcinomas *in situ* were excluded.

Breast cancer tissue microarray construction

Breast cancer samples were embedded in paraffin. A hematoxylin and eosin (HE)-stained section of each tumor block was used to identify representative tumor regions from which two 1.0-mm tissue cores were retrieved and transferred into recipient array blocks using a tissue microarrayer (UNITMA Instruments, Seoul, Korea) as previously described [15]. The TMA was composed of duplicate cores from different areas of the same tumor to compare staining patterns.

Immunohistochemistry

The TMAs were deparaffinized with xylene, gradually rehydrated with a graded ethanol series, and then washed with phosphate-buffered saline (PBS). Antigen retrieval was performed by boiling 2 groups of TMAs in 0.01 M Tris–sodium citrate buffer (pH 6.0) at 100°C for 10 min followed by a 5 min incubation at room temperature. TMAs were then incubated with nonspecific-staining blocking buffer for 20 min to quench endogenous peroxidase activity and then with anti-AR (clone AR441, Gene Tech, ShangHai, China; dilution: 1:50) or anti-BRCA1 (clone D-9, Santa Cruz Biotechnology, USA; dilution: 1:200) primary antibodies at 4°C overnight. Primary antibodies were detected by incubation with HRP-conjugated secondary antibodies for 30 min at room temperature, followed by colorimetric detection with 3,3-diaminobenzidine (DAB). The TMAs were then counterstained with Gill hematoxylin, dehydrated in an ascending ethanol series and then in xylene, and mounted with a coverslip. IHC for ER, PR, HER2 was automatically performed by the BenchMax XT IHC/ISH Staining Module (Roche) in the Department of Pathology at Fudan University Shanghai Cancer Center, according to the standard protocol.

Staining evaluation

For BRCA1, the TMAs were semi-quantitatively scored according to a staining index (SI; range 0-9) with the following formula: SI = intensity × proportion scores. the staining intensity was scored using four grades (0 negative; 1, weak; 2, moderate; and 3, strong), and proportion scores were based on the percentage of nuclear staining

positive cells (1, < 10%; 2, 10-50%; and 3, > 50%). SI > 5 was defined as BRCA1-positive staining, whereas SI < 5 was defined as negative staining [17]. The average score for duplicate cores was used for all subsequent analyses.

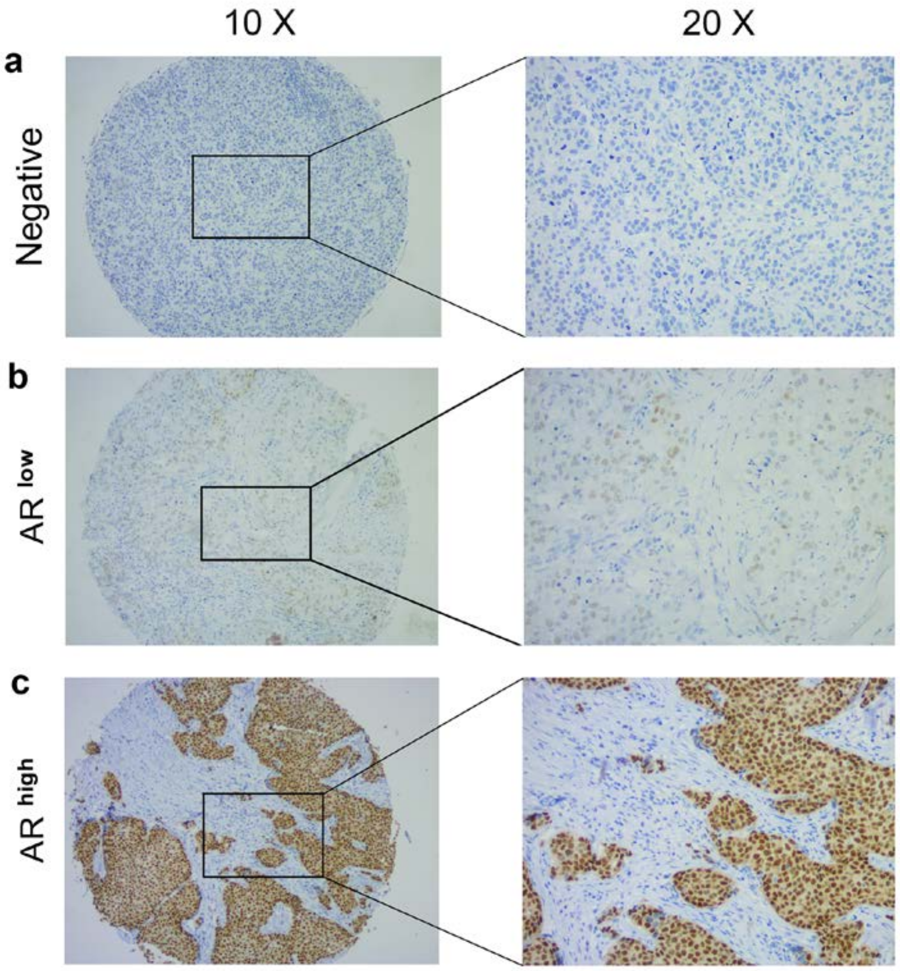

Fig. S1 Representative androgen receptor (AR) immunohistochemical staining is presented in the large (20 × magnification) and small images (10 × magnification). **a** Negative for AR. **b** Low expression for AR. **c** High expression for AR.

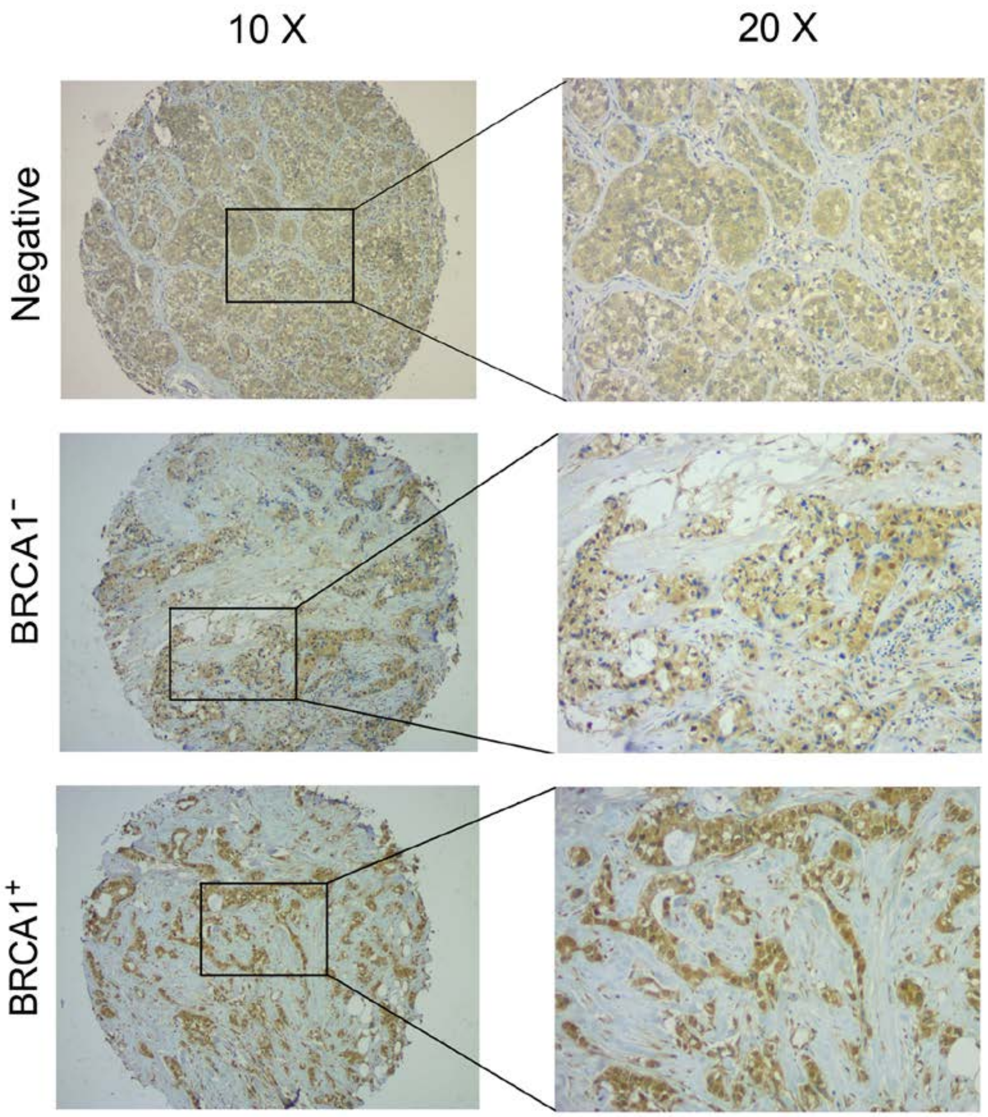

Fig. S2 Representative BRCA1 immunohistochemical staining is presented in the large (20 × magnification) and small images (10 × magnification).

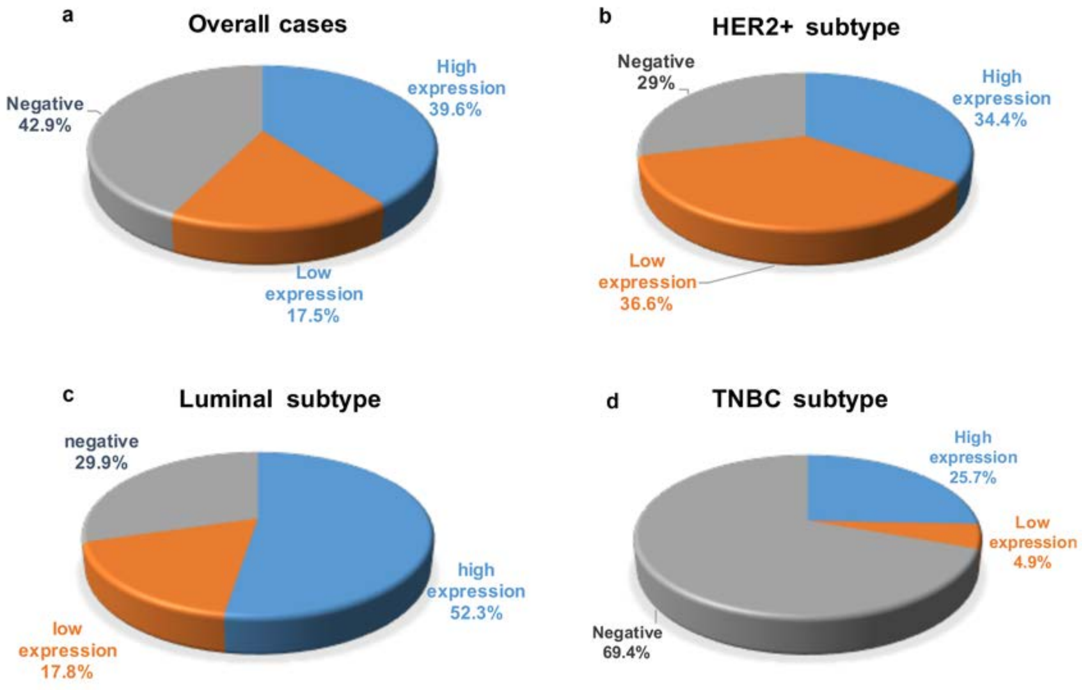

Fig. S3 Evaluation of androgen receptor (AR) expression in breast cancer. The AR expression patterns in **a**) all patients (n = 434), **b**) HER2 positive subtype(n = 93), **c**) luminal subtype (n = 197) **d**) triple negative breast cancer (TNBC) subtype (n = 144).

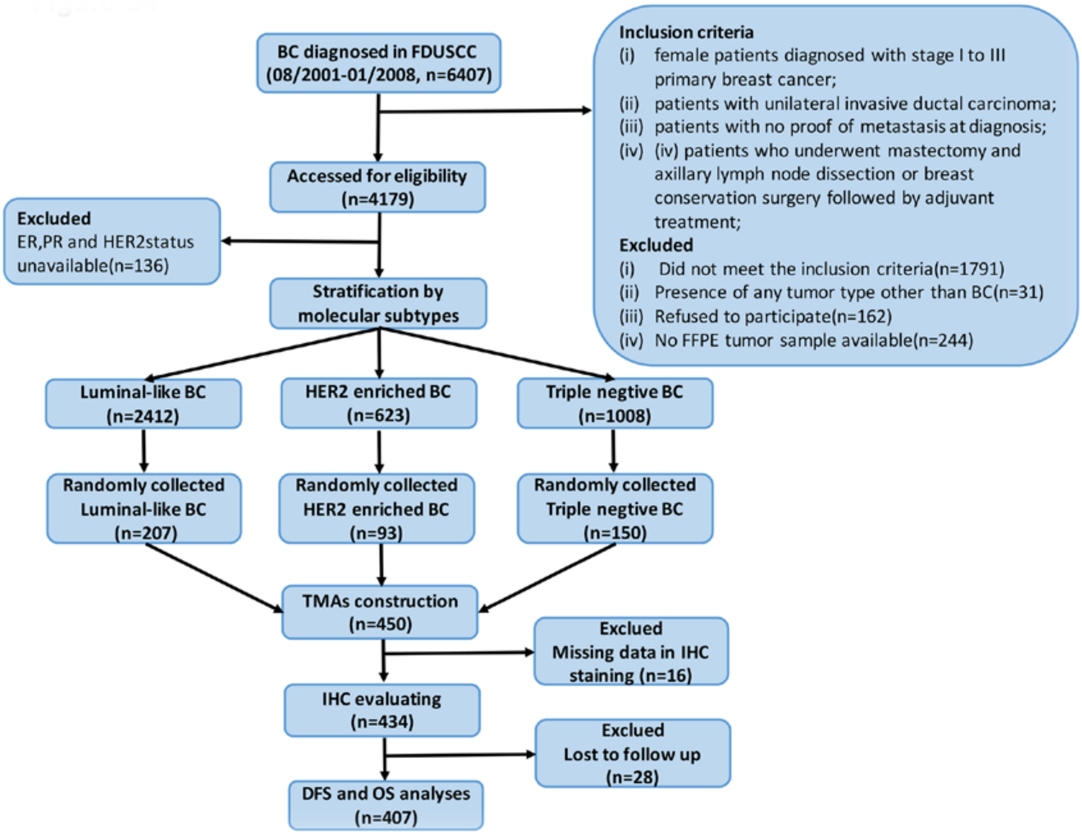

Fig. S4 A flowchart to explain the random selection process of patients in this study
